# Thermo-Responsive and Electroconductive Nano Au-PNiPAAm Hydrogel Nanocomposites: Influence of Synthesis Method and Nanoparticle Shape on Physicochemical Properties

**DOI:** 10.3390/polym16233416

**Published:** 2024-12-05

**Authors:** Nikolina Radojković, Jelena Spasojević, Zorica Kačarević-Popović, Una Stamenović, Vesna Vodnik, Goran Roglić, Aleksandra Radosavljević

**Affiliations:** 1 Vinča Institute of Nuclear Sciences, National Institute of the Republic of Serbia, University of Belgrade, Mike Petrovića Alasa 12-14, Vinča, 11351 Belgrade, Serbia; nnikolina@vin.bg.ac.rs (N.R.); zokkacpop@gmail.com (Z.K.-P.); una@vin.bg.ac.rs (U.S.); vodves@vin.bg.ac.rs (V.V.); krkljes@vin.bg.ac.rs (A.R.); 2 Faculty of Chemistry, University of Belgrade, Studentski trg 12-16, 11158 Belgrade, Serbia; groglic@chem.bg.ac.rs

**Keywords:** gold nanoparticles, PNiPAAm, hydrogel nanocomposites, thermo-responsivity, electroconductivity, thermo-switchable mechanism

## Abstract

Hydrogel nanocomposites that respond to external stimuli and possess switchable electrical properties are considered as emerging materials with potential uses in electrical, electrochemical, and biological devices. This work reports the synthesis and characterization of thermo-responsive and electroconductive hydrogel nanocomposites based on poly(*N*-isopropylacrylamide) (PNiPAAm) and gold nanoparticles (nanospheres—AuNPs and nanorods—AuNRs) using two different synthetic techniques. Method I involved γ-irradiation-induced crosslinking of a polymer matrix (hydrogel), followed by radiolytic *in situ* formation of gold nanoparticles, while Method II included the chemical synthesis of nanoparticles, followed by radiolytic formation of a polymer matrix around the gold nanoparticles. UV–Vis spectral studies revealed the presence of local surface plasmon resonance (LSPR) bands characteristic of nanoparticles of different shapes, confirming their formation and stability inside the polymer matrix. Morphological, structural, and physicochemical analyses indicated the existence of a stable porous polymer matrix, the formation of nanoparticles with a face-centered cubic structure, increased swelling capacity, and a slightly higher volume phase transition temperature (VPTT) for the hydrogel nanocomposites. Comparative electrochemical impedance spectroscopy (EIS) showed an increase in conductivity for the nano Au-PNiPAAm hydrogel nanocomposites compared to the PNiPAAm hydrogel, with a considerable rise detected above the VPTT. By reverting to room temperature, the conductivity decreased, indicating that the investigated hydrogel nanocomposites exhibited a remarkable reversible “on–off” thermo-switchable mechanism. The highest conductivity was observed for the sample with rod-shaped gold nanoparticles. The research findings, which include optical, structural, morphological, and physicochemical characterization, evaluation of the efficiency of the chosen synthesis methods, and conductivity testing, provide a starting point for future research on the given nanocomposite materials with integrated multifunctionality.

## 1. Introduction

Significant improvements in polymer nanocomposite materials with gold nanoparticles have been observed in recent decades. Because of their unique catalytic, electrical, and optical properties, these materials can be used to create a wide range of novel nanodevices. To effectively utilize the special properties of these materials, an approach for the production of metal clusters that will remain stable for long periods in polymer matrices needs to be developed.

Hydrogels, as stable three-dimensional crosslinked structures, are polymer matrices that can provide excellent platforms for the formation and stabilization of different types of nanoparticles. Due to the high porosity of hydrogels and the existence of a significant number of macro- and micropores, the nanoparticles are well dispersed within the polymer matrix and stabilized by interactions with certain groups in the polymer chains [1,2,3,4]. The main advantages of hydrogels are their excellent biocompatibility and ability to absorb and retain a large amount of fluid, which allows them to be used in applications related to biological processes. Thus, hydrogels can interact with the human body without harmful effects. Recently, hydrogels that exhibit environmentally responsive behavior and reversibly switch from a hydrophilic to hydrophobic state in response to temperature changes represent a special class of “intelligent” materials, with a wide range of applications. The most widely studied thermo-responsive polymer is poly(*N*-isopropylacrylamide) (PNiPAAm), with a volume phase transition temperature (VPTT) in the range of 30–32 °C. The closeness of the VPTT to human body temperature enables the preparation of PNiPAAm-based materials suitable for bioapplications such as human motion detection, actuators, controlled drug release, wound dressing, flexible sensors, and sensor membranes [2,5,6,7,8]. The addition of conductive components into the PNiPAAm hydrogel transforms it into a ″smart″ conductive material. Electrically conductive hydrogels can be obtained by adding metal nanoparticles, conductive polymers, carbon materials, MXene, etc., into the polymer network. As a result, PNiPAAm hydrogels can become sensitive to electrical signals, with broad application prospects in human health real-time monitoring [9,10,11]. These systems are promising materials for the growth of flexible electronics capable of responding to different external signals and can be classified as temperature sensors, chemical sensors, stress/strain sensors, humidity sensors, electric and magnetic field sensors, light sensors, etc. Flexible electronics provide several advantages over traditional rigid devices, including comparable softness to biological tissues, conformable interaction with a surface, chronic biocompatibility, and long-term wearability. Moreover, thermosensitive hydrogels can be used to fabricate actuators, microfluidic valves and controllers, flexible energy storage devices, etc. [12,13].

Metal nanoparticles show exceptional conductive properties, and Ag and Au nanoparticles are most often used to synthesize conductive hydrogels because they also possess additional biological functions, such as antibacterial and photodynamic therapy properties, respectively [9]. The choice of a suitable synthesis procedure for the preparation of hydrogel nanocomposites is the most relevant, especially if bioapplications are planned. Numerous studies have tested and optimized various methods [14,15]. Growing awareness of environmental protection has led to the development of eco-friendly approaches to the synthesis process and, accordingly, radiolytic synthesis (using γ-irradiation) was employed in this investigation to produce hydrogel nanocomposites [16,17,18]. The principle of γ-irradiation-induced synthesis is based on the biologically harmless and biocompatible radiolytic products of water, allowing polymerization and crosslinking of polymers as well as *in situ* synthesis of metal nanoparticles without any additional chemical agents. One of the most important advantages is the possibility of synthesis and sterilization of products in one technological step, which is an essential prerequisite for potential biomedical applications [19,20,21,22].

In this work, thermo-responsive and electroconductive nano Au-PNiPAAm hydrogel nanocomposites with differently shaped incorporated gold nanoparticles were produced by two different synthesis approaches. The main goals were to prove the temperature-dependent electrical conductivity of hydrogel nanocomposites and to show a reversible thermo-switchable mechanism. A detailed optical, structural, morphological, and physicochemical characterization enables the determination of key parameters required for the synthesis of systems with certain properties, thus improving the performance of the final products to meet the requirements of potential applications. The investigated nano Au-PNiPAAm hydrogel nanocomposites represent the starting point for developing more complex systems that, for example, can be used as photoactuators, i.e., systems that demonstrate a photo-thermo-mechanical effect.

## 2. Materials and Methods

### 2.1. Materials

*N*-isopropylacrylamide (NiPAAm), hydrogen tetrachloroaurate (III) trihydrate (HAuCl_4_ × 3H_2_O), trisodium citrate (Na_3_C_6_H_5_O_7_), sodium borohydride (NaBH_4_, 99%), silver nitrate (AgNO_3_), ascorbic acid (C_6_H_8_O_6_), and sulfuric acid (H_2_SO_4_) were purchased from Sigma Aldrich (Hamburg, Germany); cetyltrimethylammonium bromide (CTAB, C_19_H_42_BrN) was purchased from Fluka (Taufkirchen, Germany); while 2-propanol ((CH_3_)_2_CHOH) was obtained from Merck (Rahway, NJ, USA). All substances were commercial products of the highest available purity and were used without further purification. Water from a Millipore Milli-Q system (Millipore Corporation, Cork, Ireland), which corresponds to four times greater purity than distilled water, was used in all experiments. Before γ-irradiation (^60^Co source), the solutions were saturated with argon gas (Messer Tehnogas, 99.5% purity) (Belgrade, Serbia) to remove oxygen.

### 2.2. Chemical Synthesis of Gold Nanospheres and Gold Nanorods

The colloidal dispersion of gold nanospheres (AuNPs) was prepared by a simple process of Au^3+^ ion reduction by sodium citrate as a reducing and stabilizing agent [23]. The solution of HAuCl_4_ × 3H_2_O (1 × 10^−3^ mol/dm^3^, 200 mL) was stirred and heated in a round-bottomed flask fitted with a reflux condenser. After the solution reached the boiling point, Na_3_C_6_H_5_O_7_ (38.8 × 10^−3^ mol/dm^3^, 20 mL) was rapidly added, and the color of the solution changed, almost instantly, from yellow to burgundy due to the formation of AuNPs. The colloidal dispersion was boiled and stirred for an additional 15 min and then cooled down to room temperature with continuous stirring. After centrifugation (10 min, 12,000 rad/min) and reducing the volume of AuNPs to 2 mL, their concentration in the precipitate, determined by inductively coupled plasma-atomic emission spectrometry (ICP-AES), was found to be 0.06 mol/dm^3^.

On the other hand, the colloidal dispersion of gold nanorods (AuNRs) was prepared by a two-step synthesis procedure: formation of Au seed followed by a chemical reduction process [24]. To a mixture of CTAB (0.2 mol/dm^3^, 5 mL) and HAuCl_4_ × 3H_2_O (0.5 mol/dm^3^, 5 mL), an ice-cooled aqueous solution of NaBH_4_ (0.01 mol/dm^3^, 0.6 mL) was added, and the light yellow-brownish color of the solution indicated the formation of Au seed (kept at room temperature at least for 2 h before use). For the chemical reduction synthesis of AuNRs, CTAB solution (0.2 mol/dm^3^, 0.5 mL) was mixed with AgNO_3_ (4 × 10^−3^ mol/dm^3^, 0.25 mL) and HAuCl_4_ × 3H_2_O (1 × 10^−3^ mol/dm^3^, 5 mL) solutions. Immediately after adding ascorbic acid (0.778 × 10^−3^ mol/dm^3^, 70 mL), the yellow solution became colorless, and 10 mL of Au seed was added. Within 20 min, the color of the reaction mixture gradually changed from light to dark pink and finally to dark violet/blue, indicating AuNR formation, i.e., aging due to their growth along the longitudinal axis. Before their use, there was a need for the removal of excess CTAB. To the whole volume of as-prepared AuNRs, 25 mM NaBH_4_ was added and dissolved [24]. After resting for 1 h, the solution was centrifuged (10 min, 12,000 rad/min). Desorbed CTAB molecules were removed with the supernatant, while the AuNR precipitate was re-dispersed in 2 mL of water. The concentration of Au in this precipitate, determined by ICP-AES, was found to be 0.05 mol/dm^3^.

### 2.3. Synthesis of PNiPAAm Hydrogel

An aqueous solution of NiPAAm (10 wt%) was saturated with argon for 30 min, poured into molds consisting of two glass plates separated by a rubber spacer, and exposed to γ-irradiation at a dose rate of 0.3 kGy/h up to an absorbed dose of 25 kGy to induce radical polymerization and crosslinking. After irradiation, the hydrogel was cut into discs, dried in a vacuum oven at 25 °C to constant weight, and subjected to extraction with water to remove uncrosslinked polymer and/or residual monomer.

### 2.4. Synthesis of Nano Au-PNiPAAm Hydrogel Nanocomposites

In this work, the nano Au-PNiPAAm hydrogel nanocomposites were produced by two different approaches: Method I—radiolytic *in situ* formation of gold nanoparticles inside the previously obtained PNiPAAm hydrogel, and Method II—γ-irradiation-induced polymerization and crosslinking of NiPAAm monomer in the presence of chemically produced gold nanoparticles.

Method I: To obtain AuNPs-PNiPAAm nanocomposites, PNiPAAm hydrogel discs (diameter of 10 mm and thickness of 4 mm) were immersed into argon-saturated solutions containing Au^3+^ ions (1 × 10^−4^ mol/dm^3^ and 2.5 × 10^−4^ mol/dm^3^) and 2-propanol (0.2 mol/dm^3^). Subsequently, to obtain AuNRs-PNiPAAm nanocomposites, PNiPAAm hydrogel discs were immersed into argon-saturated solutions containing Au^3+^ ions (1 × 10^−4^ mol/dm^3^ and 2.5 × 10^−4^ mol/dm^3^), CTAB (0.2 mol/dm^3^, 8 mL), AgNO_3_ (1 × 10^−2^ mol/dm^3^, 0.24 mL), diluted H_2_SO_4_ (0.3 mL), ascorbic acid (0.1 mol/dm^3^, 0.24 mL), 2-propanol (0.2 mol/dm^3^), and distilled water (12 mL). All samples were left to swell for 48 h in the dark and then exposed to γ-irradiation at the dose rate of 10 kGy/h up to absorbed doses of 0.7 kGy and 1.75 kGy for lower and higher Au^3+^ ion concentrations, respectively.

Method II: Chemically prepared AuNPs and AuNRs (Section 2.2) were mixed with 10 wt% NiPAAm solution to obtain concentrations of gold nanoparticles of 1 × 10^−4^ mol/dm^3^ and 2.5 × 10^−4^ mol/dm^3^. These solutions were saturated with argon, poured into glass molds, and exposed to γ-irradiation at the dose rate of 0.3 kGy/h up to an absorbed dose of 25 kGy to induce radical polymerization of NiPAAm and formation of a crosslinked polymer network around the nanoparticles.

### 2.5. Methods of Characterization

*Optical properties*. The absorption spectra of the Au colloidal dispersions and nano Au-PNiPAAm hydrogel nanocomposites were recorded using an Evolution 600 UV–Vis spectrophotometer (Thermo Fisher Scientific, Waltham, MA, USA) in the wavelength range of 300–900 nm. The Au colloidal dispersions were recorded in quartz cuvettes (optical path 1 cm), while the nanocomposite samples were recorded in the post-synthesis state by placing the hydrogel nanocomposites (thickness 0.1 mm–0.5 mm) directly in the optical path of light.

*Transmission electron microscopy (TEM).* To determine the size and morphology of the prepared gold nanoparticles in the colloidal dispersions, TEM investigation was performed using a JEOL JEM-2100 LaB6 instrument operated at 200 KV (JEOL company, Peabody, MA, USA). TEM images were acquired with a Gatan Orius CCD camera at 2× binning. A drop of each solution was deposited on a carbon-coated copper grid, allowed to dry at room temperature, and then examined under an electron microscope.

*Field emission scanning electron microscopy (FE-SEM).* The internal morphology of the synthesized systems was examined using field emission scanning electron microscopy (FEI Scios2, Dual Beam system, Thermo Fisher Scientific, Waltham, MA, USA). After synthesis, samples were frozen at −20 °C for two days before being lyophilized using a Martin Christ Alpha 1-2 LDplus freeze dryer (Osterode am Harz, Germany). The lyophilization procedure was carried out for 24 h at a temperature of –32 °C and a vacuum of 0.310 mbar. Prior to the SEM analysis, the freeze-dried samples were fractured and covered with thin gold layer (around 15 nm) using a LEICA SCD005 nebulizer (Wetzlar, Germany).

*Inductively coupled plasma-atomic emission spectrometry (ICP-AES).* The content of Au in the colloidal dispersions was determined using an ICP-AES Spectroflame 17 (Spectro-Analytical Instruments, Kleve, Germany) operated at 4 MHz. Samples of both Au colloidal dispersions (0.1 mL of AuNPs and AuNRs) were separately dissolved in 0.9 mL of a mixture of concentrated nitric and hydrochloric acid to prepare for ICP analysis.

*Gel content.* After the completion of the crosslinking process and formation of the polymer network, the obtained PNiPAAm hydrogels and nano Au-PNiPAAm hydrogel nanocomposites were cut into discs and dried at room temperature to constant weight. Then, the samples were immersed in distilled water, which was changed daily for a week, to remove unreacted substances. The extracted hydrogels were dried again, and the gel content was calculated as follows:

(1) $$W_{g}\;(\%)=\frac{m_{ae}}{m_{be}}\cdot 100$$

where *m_ae_* and *m_be_* are the weights of the dry gels after and before extraction, respectively. All measurements were performed in triplicate.

*Swelling studies.* Swelling of the hydrogel and nanocomposite samples was gravimetrically monitored in distilled water at 25 °C. The xerogel discs (diameter 5 mm, thickness 1.5 mm) were immersed in water and measured at predetermined time intervals until a constant mass was reached. The degree of swelling was calculated according to the following equation:

(2) $$SD=\frac{m_{t}-m_{0}}{m_{0}}$$

where *m_t_* is the weight of swollen hydrogel at predetermined time intervals, and *m*_0_ is the initial weight of the xerogel. The swelling tests were carried out in triplicate and the results are expressed as mean values.

*Determination of volume phase transition temperature (VPTT).* The temperature sensitivity of the synthesized hydrogels and VPTT values were determined gravimetrically in the temperature range of 12–48 °C. After immersion in water at a specific fixed temperature, the hydrogels were left to swell to equilibrium and then weighed. The samples were then re-equilibrated in distilled water at another predetermined temperature and their swollen weight was again determined. To ensure reliability, all obtained results were expressed as the average value of three independent measurements.

*Deswelling studies.* Deswelling kinetic experiments were carried out by immersing pre-weighed fully swollen hydrogels in distilled water at 48 °C (above the VPTT). During the deswelling process, the weight of the hydrogels was measured at predetermined time intervals until they approached a constant value. Water retention was defined as follows:

(3) $$WR=\frac{m_{t}-m_{col}}{m_{eq}-m_{col}}$$

where *m_t_* is the weight of the hydrogel at predetermined time intervals during the deswelling process, *m_eq_* is the weight of the equilibrium swollen hydrogel at the beginning, and *m_col_* is the weight of the collapsed hydrogel at the end of the deswelling process. To ensure reliability, all obtained results were expressed as the average value of three independent measurements.

*X-ray diffraction (XRD).* The crystallographic structure of the nanoparticles was examined on a Bruker D8 Advance diffractometer (Cu K_α1_ radiation, λ = 0.1541 nm) (Bruker, Billerica, MA, USA). Diffractograms were recorded in the 2θ range between 10° and 85°, with an exposure time of 10s and a step of 0.05°.

*Fourier-transform infrared (FTIR) analysis.* To examine the molecular structure of the hydrogel matrix and its interaction with gold nanoparticles, FTIR analysis of PNiPAAm hydrogels and the corresponding hydrogel nanocomposites was performed using a Nicolet 380 Spectrophotometer in the 4000–400 cm^−1^ range, with the addition of attenuated total reflection (ATR) mode (Thermo Fisher Scientific, Waltham, MA, USA). The examined samples were recorded in the xerogel state after being dried at ambient temperature to a consistent mass.

*Electrochemical measurements.* The electrical conductivity of the hydrogel nanocomposites was measured using electrochemical impedance spectroscopy (EIS) with a Gamry Reference 600 potentiostat, while the impedance spectra were analyzed using the Gamry Instruments Echem Analyst fitting procedure (Gamry Instruments, Warminster, PA, USA). An electrochemical cell employing two platinum electrodes (one serving as the working electrode and the other as the reference electrode) was utilized for these experiments. EIS was conducted across a frequency range of 10 Hz to 100 kHz with a potential amplitude of 10 mV relative to the open circuit potential. Measurements were performed on samples in their initial state, i.e., at room temperature (25 °C), then thermostated for 60 min at 40 °C and measured. After that, the hydrogels were cooled again to room temperature for 60 min, and the EIS procedure was repeated once more. All measurements were performed in a triplicate.

## 3. Results and Discussion

### 3.1. Synthesis and Optical Features of Nano Au-PNiPAAm Hydrogel Nanocomposites

**Figure 1:**
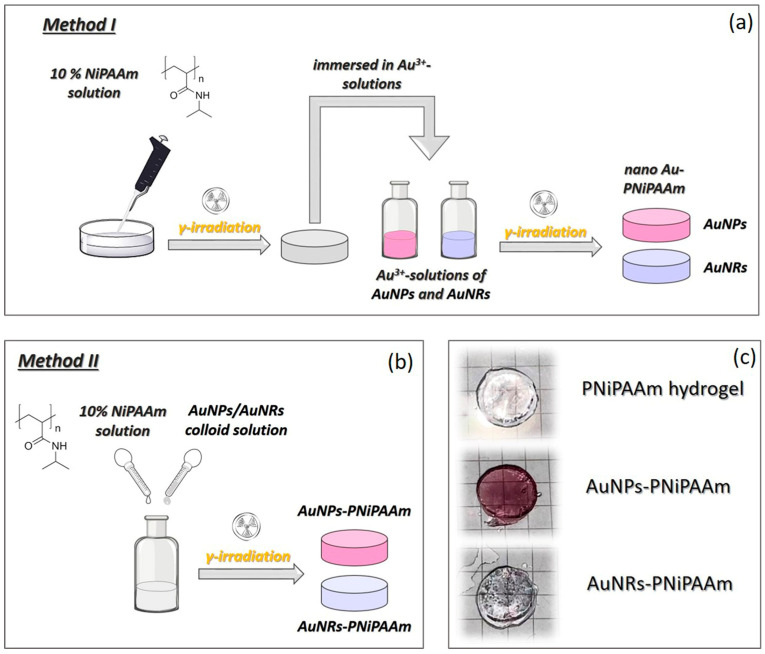
Schematic representation of synthesis: Method I (**a**), Method II (**b**), and photographs of nano Au-PNiPAAm hydrogel nanocomposites obtained by Method II (**c**).

In this investigation, two different approaches to the synthesis of nano Au-PNiPAAm hydrogel nanocomposites were applied. The first method (Method I) involved γ-irradiation-induced *in situ* synthesis of gold nanoparticles of different shapes in a previously prepared polymer matrix (hydrogel) (Figure 1a). The other approach involved the chemical synthesis of Au nanoparticles, followed by γ-irradiation-induced formation of a polymer matrix around them (Method II) (Figure 1b).

The difference between these two methods is that, in Method I, the reducing species for nanoparticle formation were generated *in situ* (products of water radiolysis under γ-irradiation), whereas in Method II, the reducing agent was introduced into the system from an external source (chemical substances that were added to the solution) [20].

#### 3.1.1. γ-Irradiation-Induced *In Situ* Synthesis—Method I

*Polymer matrix (hydrogel) preparation.* When aqueous monomer/polymer solutions are exposed to γ-irradiation, the primary process that occurs is the radiolysis of water and the formation of several primary species. The main reactive radicals among these basic radiolysis products are OH^●^ radicals, as oxidizing, hydrated electrons (e_aq_^−^), and H^●^ radicals, as the reducing type (Equation (4)). It is well known that the radiation crosslinking of monomer and polymer molecules is mainly induced by OH^●^ radicals in an aqueous medium (Equation (5)) [1,18,25,26]:

(4) $$H_{2}O\overset{\gamma }{\to }e_{\mathrm{aq}}^{-},\;{\mathrm{OH}}^{•},\;H^{•},\;{H_{3}O}^{+},\;H_{2},\;H_{2}O_{2}$$

(5) $$\mathrm{NiPAAm}+{\mathrm{OH}}^{•}\to {\mathrm{NiPAAm}}^{•}+H_{2}O$$

In an argon-saturated aqueous solution, hydroxyl radicals attack NiPAAm molecules, causing breaking of the –C=C– double bond, generating free radicals that react with each other, and thus producing macroradicals. Upon further irradiation, recombination of the generated macroradicals initiates the polymerization process, leading to the formation of a stable crosslinked 3D polymer network (i.e., a hydrogel). The porous structure of hydrogels and the presence of large hydrated spaces between the polymer chains make them suitable nanoreactors for the formation, growth, and stabilization of nanoparticles [1,27,28].

*In situ synthesis of Au nanoparticles inside the previously prepared polymer matrix*. To prove the potential of the polymer matrix for nanoparticle formation and stabilization inside the network, PNiPAAm hydrogels were swollen to equilibrium in a solution containing Au^3+^ ions and then exposed to γ-irradiation. Considering that these are aqueous solutions of gold ions, the process that takes place after γ-ray exposure is the radiolysis of water and the formation of radical species. Several studies have established the interaction of Au^3+^ with reducing species leading to the creation of Au^0^ clusters and subsequently Au nanoparticles (Equations (6) and (7)) [29,30]:

(6) $${\mathrm{Au}}^{\mathrm{III}}\to {\mathrm{Au}}^{\mathrm{II}}\overset{{\mathrm{Au}}^{\mathrm{II}}}{\to }{\mathrm{Au}}_{2}^{\mathrm{II}}\to {\mathrm{Au}}^{\mathrm{III}}+{\mathrm{Au}}^{I}\overset{{\mathrm{Au}}_{n}}{\to }{\mathrm{Au}}^{I}\;{\mathrm{Au}}_{n}\to {\mathrm{Au}}_{n+1}$$

(7) $${\mathrm{Au}}^{\mathrm{III}}\overset{\mathrm{Au}}{\to }{\mathrm{Au}}^{\mathrm{II}}+{\mathrm{Au}}^{I}$$

Considering that the process of nanoparticle formation involves the reduction of Au^3+^ ions to zero-valent Au atoms (Au^0^), unwanted oxidizing species (OH^●^ radicals) that can negatively affect the system balance must be removed. This can be achieved by adding radical scavengers (2-propanol) that have an affinity to chemically interact with undesired species, inactivating or eliminating them from the system (Equation (8)) [1,18,31,32]:

(8) $${\left({\mathrm{CH}}_{3}\right)}_{2}\mathrm{CHOH}+{\mathrm{OH}}^{•}\to {\left({\mathrm{CH}}_{3}\right)}_{2}C^{•}\mathrm{OH}+H_{2}O$$

Different shapes of gold nanoparticles were created by a multistep reduction process of gold ions using substantially reduced hydrated electrons, 2-propanol radicals, and polymer radicals. Because the binding energy of two atoms is greater than the atom–solvent and atom–ligand bond energies, the resulting Au^0^ atoms tend to dimerize, resulting in the progressive growth and creation of spherical metal clusters. It should be emphasized that, in the case of nanorod synthesis, it was necessary to add CTAB to the Au^3+^ ion solution as a surfactant to induce anisotropy. These additives first adsorb on the precursor metal ions, thus creating anisotropic confinements by selective passivation of certain facets to induce and maintain anisotropic crystal growth. This approach enables the generation of specially shaped and highly crystalline nanostructures with a high yield [1,20,31]. Due to the porous structure of the polymer matrix (PNiPAAm hydrogel), the produced nanoparticles were uniformly dispersed throughout the matrix and stabilized by interactions with the polymer chains [33].

**Figure 2:**
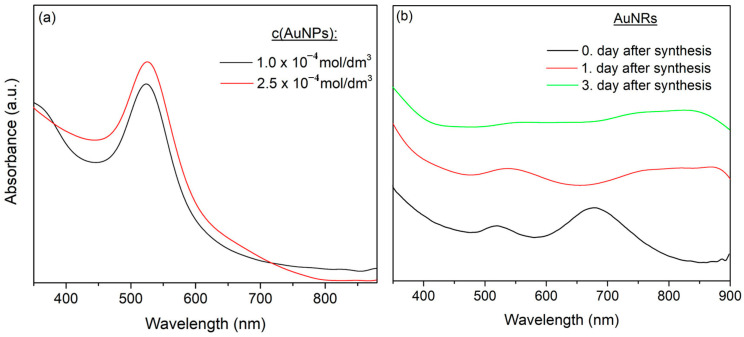
UV–Vis absorption spectra of AuNPs-PNiPAAm (**a**) and AuNRs-PNiPAAm (**b**) hydrogel nanocomposites obtained by Method I.

In order to investigate the ability of the PNiPAAm polymer network to stabilize and control the formation of *in situ* synthesized gold nanoparticles with different sizes and shapes, UV–Vis spectroscopy was used. The first confirmation of the successful incorporation of gold nanoparticles within the polymer matrix was the change in color of the hydrogel samples after the synthesis procedure (Figure 1c). The hydrogel nanocomposites with AuNPs were light burgundy, while those with AuNRs were light blue. The optical properties of metallic nanoparticles arise from the collective vibration of conduction electrons in the nanoparticles when they interact with electromagnetic radiation. The incident light’s electric field causes the formation of a dipole in nanoparticles, with the restoring force attempting to compensate for it, resulting in a unique resonance wavelength in the visible region, i.e., the localized surface plasmon resonance (LSPR) band. The obtained UV–Vis absorption spectra of the AuNPs-PNiPAAm hydrogel nanocomposites (Figure 2a) revealed the presence of LSPR bands around 520 nm, indicating the formation of spherical gold nanoparticles [24,34].

Due to the difference in electron resonance oscillations along the longer and shorter axes, AuNRs have two extinction peaks, transversal oscillation around 530 nm and longitudinal oscillation around 760 nm [24]. UV–Vis spectroscopic analysis revealed the presence of peaks at the expected wavelength values when measurements were taken right after synthesis (Figure 2b). However, contrary to expectations, a few days after synthesis, the tested sample became colorless and the characteristic LSPR bands of AuNRs (transversal and longitudinal oscillations) in the absorption spectra were no longer visible due to the disintegration or dissolution of the nanorods.

The obtained results clearly indicated that, in the examined case and under the stated experimental conditions, the radiation chemical method using γ-rays produced unstable AuNRs that required either further stabilization or the use of another acceptable synthesis method. To overcome this problem and produce AuNRs-PNiPAAm hydrogel nanocomposites, Method II was used, which involved embedding chemically synthesized AuNRs into a polymer matrix.

#### 3.1.2. Chemical Method of Synthesis—Method II

**Figure 3:**
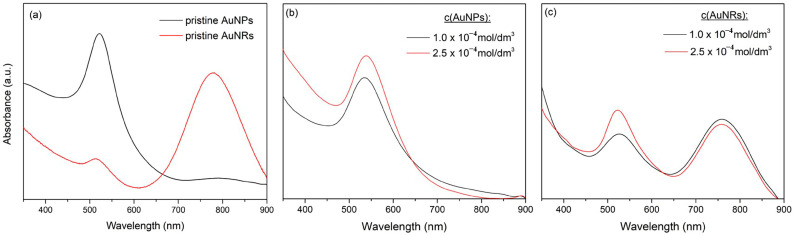
UV–Vis absorption spectra of chemically synthesized colloidal dispersion of AuNPs and AuNRs (**a**), AuNPs-PNiPAAm (**b**), and AuNRs-PNiPAAm (**c**) hydrogel nanocomposites obtained by Method II.

*Preparation of colloidal dispersions of AuNPs and AuNRs.* A chemical method of synthesis was used to obtain colloidal dispersions of Au nanoparticles with different shapes, nanospheres and nanorods. The colloidal dispersions of AuNPs were prepared by a simple process of reduction of Au^3+^ ions with sodium citrate as a reducing and stabilizing agent. The formation of nanospheres could be visually observed by a change in the reaction mixture color from yellow to burgundy. Furthermore, AuNRs were synthesized with the addition of CTAB as a surfactant to induce anisotropy, i.e., growth along the longitudinal axis. The color of the reaction mixture gradually changed from light to dark pink and finally to dark violet/blue, indicating nanorod formation [24]. The characteristic LSPR bands for both pristine AuNPs and AuNRs are clearly visible in the absorption spectra presented in Figure 3a.

The UV–Vis absorption spectra recorded frequently over three months (figure not presented here) indicated that the formed Au nanoparticles were stable over a longer period. As a result, it was established that this method of synthesis was more suitable for the formation of stable nanorods, and these samples were further evaluated.

*Preparation of AuNPs-PNiPAAm and AuNRs-PNiPAAm hydrogel nanocomposites.* Chemically prepared AuNPs and AuNRs were mixed with NiPAAm solution and exposed to γ-rays to induce radical polymerization and crosslinking in order to obtain a polymer network around the nanoparticles (Figure 1b). The obtained absorption spectra of the nano Au-PNiPAAm hydrogel nanocomposites prepared by Method II confirmed the presence of nanoparticles with different shapes inside the polymer matrix (Figure 3b,c). The characteristic LSPR band appeared at around 530 nm for the AuNPs-PNiPAAm nanocomposites, while for the AuNRs-PNiPAAm nanocomposites, the characteristic bands were positioned at around 530 nm and 760 nm for transversal and longitudinal oscillation, respectively [24].

LSPR strongly depends on the shape, size, density, inter-particle distance, size distribution, and dielectric properties of the nanoparticles as well as the substrate and/or surrounding medium. These parameters affect the movement of conducting electrons within nanoparticles and permit tuning of the LSPR wavelength region of nanoparticles [24,35]. The size of the AuNPs for pristine colloidal solution and for the AuNPs obtained by both synthesis methods was determined using Mie theory, based on the experimentally obtained UV–Vis absorption spectra [36]. In addition to the possibility of determining the size of the AuNPs (Method I), with this method it was also possible to evaluate the influence of the exposure of chemically synthesized nanospheres to γ-radiation during formation of the polymer network (Method II). When the dimension of the metal nanoparticle is smaller than the wavelength (*λ*) of incident radiation, the average radius (*r*) can be calculated from the values of full width at half maximum (FWHM) of the LSPR band, using the following equation:

(9) $$r=\frac{v_{f}}{{∆\omega }_{1/2}}$$

where *ν_f_* is the Fermi velocity for gold (1.4 × 10^8^ m/s), and Δ*ω*_1/2_ is the width of the absorption band at half height in units of angular frequency (FWHM) [18,37]. The results showed that the diameter of the pristine AuNPs was around 12 nm, while the diameters of the nanoparticles produced by Method I and Method II were around 14 nm and 15.9 nm, respectively. In both synthesis methods, changes in the initial Au^3+^ concentration had no effect on the size of the nanoparticles. This result indicated that, under the stated experimental conditions, during Method II for the synthesis of hydrogel nanocomposites (absorbed dose of 25 kGy for crosslinking of polymer matrix), a slight aggregation of incorporated nanoparticles occurred compared to a stable colloidal dispersion (pristine AuNPs). Several studies have focused on γ-irradiation effects on nanoparticle stability, including changes in their physicochemical properties, size, surface reactivity, thermal stability, etc. Different aspects like nanoparticle size and concentration, total absorbed dose, and dose rate should be considered to understand and predict the influence of γ-rays on chemically prepared nanoparticles in the process of polymer matrix formation. Previous investigations demonstrated that low doses of γ-irradiation and high doses delivered from a low-energy source (or low dose rate), however, were well tolerated by most studied nanoparticles [38]. Citrate-stabilized colloidal gold nanoparticles developed by the National Institutes of Standards and Technologies (NIST) as nanoparticle reference materials (RM 8011, 8012 and 8013) were sterilized by 32 kGy of γ-irradiation with no observed change in their physicochemical properties (including size) [38,39,40]. The obtained results undoubtedly showed that the chemically produced nanoparticles, despite a minor increase in size, were stable even after irradiation and formation of the polymer network around them.

#### 3.1.3. The Efficiency of the Chosen γ-Irradiation Method for the Crosslinking Process

To confirm that the radiolytic method was suitable and effective for the crosslinking of polymers and the formation of a stable 3D polymer network, the amount of unreacted residuals after polymer crosslinking as well as the percentage of gel were determined. Determination of the mass of unreacted substances indicated that the synthesis was complete, as the evaporation of water after the extraction left an immeasurable amount of residues. The percentage of gel content in the polymer matrix was one of the most important parameters that can be used to evaluate the efficiency of the selected method (Equation (1)) [1,32]. The obtained results for the gel content were around 98.3%, 97.6%, 97.3%, and 97.9% for the PNiPAAm polymer matrix, AuNPs-PNiPAAm nanocomposites obtained by Method I, and AuNPs-PNiPAAm and AuNRs-PNiPAAm nanocomposites obtained by Method II, respectively. Such results confirmed that the chosen method of polymer matrices crosslinking under the influence of γ-irradiation was suitable as it provided high yields without unreacted substances.

### 3.2. Morphological Properties of the Nanoparticles and Polymer Network

**Figure 4:**
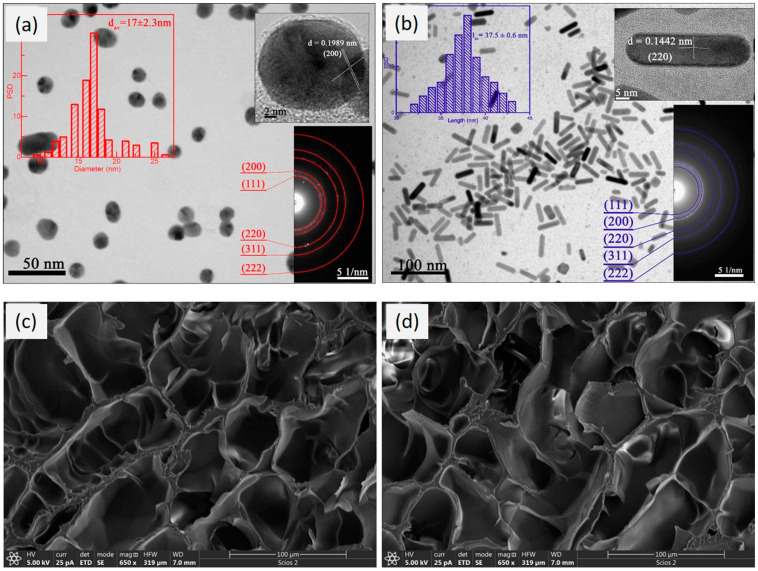
TEM micrographs of chemically synthesized AuNPs (**a**) and AuNRs (**b**), with corresponding particle size distribution (PSD, (**a**,**b**) upper-left insets), HRTEM images ((**a**,**b**) upper-right insets), and SAED ((**a**,**b**) lower-right insets), and FE-SEM micrographs of the AuNPs-PNiPAAm hydrogel nanocomposites obtained by the Method I (**c**) and AuNRs-PNiPAAm hydrogel nanocomposites obtained by the Method II (**d**).

The structural characteristics and morphology of the chemically synthesized gold nanoparticles (used in Method II) as well as the crosslinked nanocomposites were examined by TEM and FE-SEM analysis, respectively (Figure 4).

The obtained micrographs revealed the formation of spherical AuNPs with a diameter in the range between 10 nm and 28 nm, where the histogram of the nanoparticle size distribution showed that the diameter mean value was around 17 nm (Figure 4a). In addition, in Figure 4b, the formation of non-agglomerated AuNRs, uniform in size with ≈8 nm in diameter and ≈37.5 nm in length (an aspect ratio ≈ 4.7) can be seen. Furthermore, in the HRTEM images (Figure 4a,b, insets) of single particles, well-defined crystallographic planes with interplanar distances of 0.1989 nm for AuNPs and 0.1442 nm for AuNRs were observed, corresponding to the (200) and (220) planes, respectively (JCPDS card no. 04-0784). These planes, together with (111), (222), and (311) visible in the selected area electron diffraction (SAED) patterns (Figure 4a,b, insets) displayed the characteristic crystallographic face-centered cubic (*fcc*) structure of Au [24].

The internal morphology of the investigated nano Au-PNiPAAm hydrogel nanocomposites was examined by cross-sectional analysis of previously lyophilized samples using scanning electron microscopy (FE-SEM), and representative micrographs are depicted in Figure 4c,d. As previously examined and demonstrated, γ-irradiation-induced crosslinking of PNiPAAm resulted in the formation of a stable and porous 3D polymer matrix [1]. The obtained FE-SEM micrographs of the hydrogel nanocomposites synthesized by both methods showed the expected porous structure with similar morphology, with clearly visible connected pores and smooth and non-porous walls [32,41]. The gold nanoparticles were not visible in the micrographs due to the limitations of the instrument and the recording procedure.

### 3.3. Physicochemical Characterization

As already mentioned, the most recognizable properties of PNiPAAm-based hydrogels are their ability to absorb a large amount of fluid and sensitivity to changes in the surrounding temperature. Considering that, the physicochemical characterization included examination of the swelling and deswelling processes as well as determination of the VPTT values of the investigated systems.

**Figure 5:**
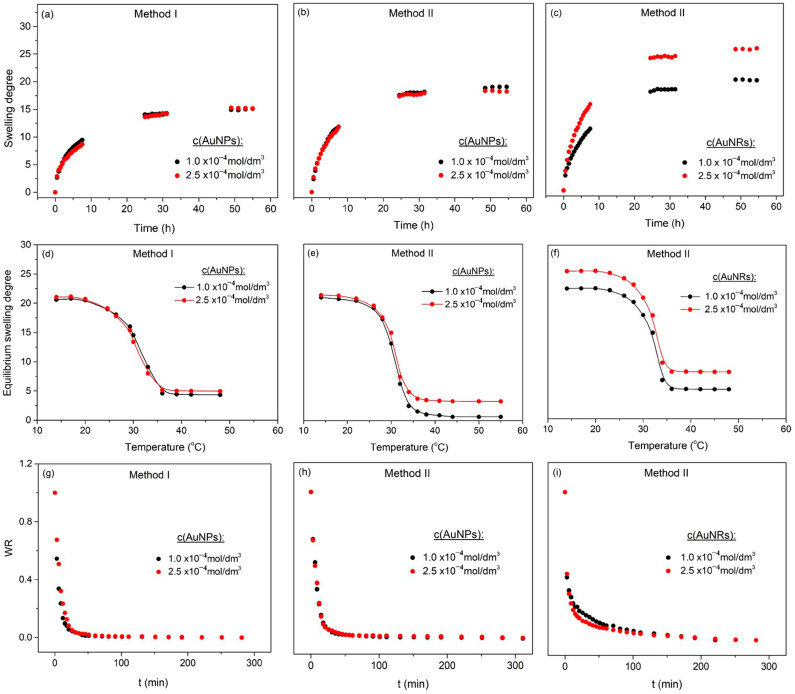
The swelling behavior (**a**–**c**), temperature dependence of *SD_eq_* (**d**–**f**), and deswelling curves (**g**–**i**) of AuNPs-PNiPAAm and AuNRs-PNiPAAm hydrogel nanocomposites.

Swelling experiments were performed to investigate the PNiPAAm-based hydrogel systems’ behavior in distilled water at ambient conditions. The swelling properties of hydrogels are highly dependent on the nature and structure of the polymer network, crosslinking density, polymer chain flexibility, environmental conditions under which the application is designed, etc. When a dry sample (xerogel) begins to swell, water molecules first hydrate the most polar hydrophilic groups. This causes the polymer chains to expand and the hydrophobic groups to come into contact with the water molecules until the equilibrium degree of swelling is reached (Figure 5a–c). The swelling capacity determined by Equation (2) is a property that affects all other hydrogel parameters, as well as its application possibilities [1].

The equilibrium swelling degree (*SD_eq_*) was also determined according to Equation (2), using the mass at the end of the swelling process, i.e., in equilibrium (*m_eq_*). In addition to the swelling capacity, the related kinetic parameters should be considered to provide a full description of the swelling process. In this study, a power law approach was used to calculate the kinetic parameters of diffusion, as follows:

(10) $$\frac{SD}{{SD}_{eq}}=kt^{n}$$

where *k* is the kinetic rate constant, while *n* is a characteristic exponent describing the mechanism of fluid transport into the polymer matrix. The logarithmic form of Equation (10) gives a linear dependence on the initial degree of swelling (*SD/SD_eq_* ≤ 0.6), so the values of *n* can be calculated from the slope. Depending on the value of the exponent, there are several diffusion models: (i) Fickian diffusion (*n* ≤ 0.5), where the rate of diffusion is significantly slower than the rate of the polymer chains relaxation; (ii) non-Fickian diffusion (0.5 < *n* <1), where the effects of diffusion and relaxation of polymer chains are comparable; and (iii) type II diffusion (*n* = 1), where the rate of diffusion is greater than the rate of polymer chain relaxation [1,42,43]. By monitoring the swelling kinetics starting from Fick’s law, it was possible to determine the diffusion coefficient (*D*) for the PNiPAAm hydrogels and nano Au-PNiPAAm hydrogel nanocomposites using the following equation:

(11) $$D={\left(\frac{k\;\cdot \;\pi \;\cdot \;r^{2}}{4}\right)}^{1/n}$$

where *r* is the radius of the xerogels [44].

The results presented in Table 1 clearly show that the presence of Au nanoparticles in the polymer network affected the hydrogel swelling behavior. Observing the results obtained for all investigated systems, it could be concluded that the incorporation of nanoparticles in polymer matrices led to an increase in swelling capacity, regardless of their shape or concentration. The exception was AuNRs produced by Method I, which did not appear to be stable; therefore, the swelling capacity of these samples was nearly comparable to that of the PNiPAAm polymer matrix without nanoparticles. In general, the presence of nanoparticles caused polymer network expansion and an increase in the free space between polymer chains, thus enhancing the fluid absorption capacity. The increase in swelling capacity was more noticeable in the case of hydrogel nanocomposites with nanorods, compared to systems with nanospheres. Due to their shape, nanorods are more difficult to pack between polymer chains, resulting in the formation of larger pores, and therefore to an increase in swelling capacity. In addition, it was evident that the PNiPAAm hydrogel and nano Au-PNiPAAm hydrogel nanocomposites showed non-Fickian diffusion, indicating that both diffusion and polymer chains relaxation processes controlled fluid transport. This behavior was more pronounced for nanocomposite samples, especially for the AuNRs-PNiPAAm synthesized by Method II. Therefore, as expected, the same samples also showed the highest values for the diffusion coefficient, whereby the influence of the concentration of the incorporated nanoparticles could also be observed. Nanocomposites with higher nanoparticle concentrations exhibited a higher diffusion coefficient, both for AuNPs-PNiPAAm and AuNRs-PNiPAAm.

The VPTT of the investigated samples was determined through the equilibrium swelling degree values in the temperature range from 12 to 48 °C, and the temperature dependence of *SD_eq_* is shown in Figure 5d–f. PNiPAAm is the most studied thermo-responsive polymer, which undergoes a sharp coil–globule transition as a response to temperature variations in the surrounding environment. Below the VPTT, PNiPAAm exhibits an extended hydrophilic chain conformation, causing water uptake and swelling of hydrogels. Increasing the temperature above VPTT results in an imbalance between hydrophilic and hydrophobic polymer–water interactions and the breaking of hydrogen bonds, causing the leaching of water and collapse of the hydrogel [1,2,45]. The closeness of the VPTT to human body temperature makes PNiPAAm one of the most studied thermo-responsive polymers, which has wide use in many applications, with special reference to the biomedical field. However, it is important to note that the described hydrogel has a wide range of applications in areas where the synergistic effect of the thermo-responsivity of the polymer and the different characteristic properties of the incorporated metal NPs can be utilized. For example, the addition of conductive components into the PNiPAAm hydrogel network transforms it into a conductive hydrogel and, as a result, the PNiPAAm hydrogel may become electrically sensitive, considerably expanding its application [9]. Therefore, in order to accurately prepare samples for potential applications, it is essential to evaluate the impact of nanoparticle incorporation into the polymer matrix on the VPTT. As can be seen in Table 1, the VPTT slightly increased in the presence of nanoparticles, regardless of whether they were obtained by Method I or Method II. Bearing in mind that during the volume phase transition, hydrogen bonds are broken and the hydrogel collapses, the presence of nanoparticles affects those processes in two ways: they act as a physical barrier to the motion and integration of polymer chains, as well as the release of water, and they increase the number of hydrogen bonds they form with specific polymer groups. Due to the mentioned processes, the phase transition temperature was shifted to higher values in the case of the nano Au-PNiPAAm hydrogel nanocomposites. The largest change in phase transition to higher values was observed for nanocomposites with chemically prepared nanorods. Since nanorods occupy more space and are more difficult to insert between polymer chains, the geometry of the nanoparticles has a greater influence. Moreover, in this case, the polymer matrix was prepared in the presence of nanoparticles, resulting in larger pores. The concentration of incorporated nanoparticles, on the other hand, has no significant effect on the phase transition temperature [1,6].

Deswelling studies were performed in order to investigate the influence of the incorporated Au nanoparticles on the hydrogel nanocomposite response at temperatures above the VPTT. To investigate the deswelling kinetics, samples swollen to equilibrium in deionized water at ambient temperature were fast transferred into hot distilled water (48 °C). The water retention (*WR*) was calculated using Equation (3) to evaluate their deswelling capacity, and the results are presented in Figure 5g–i. It is evident that the amounts of water absorbed by all the examined hydrogels were reduced almost immediately during initial deswelling and then gradually decreased until they approached the equilibrium amounts. The deswelling rate (*r_d_*) is assumed to follow first-order kinetics and is described by the following equation:

(12) $$r_{d}=-\left({dq}_{t}/d_{t}\right)=K_{d}(q_{t}-q_{md})$$

To investigate the deswelling kinetics quantitatively, a semi-logarithmic plot of first-order rate analysis, given by Equation (13), was used to fit the time dependence of the deswelling, as follows:

(13) $$ln\left[\frac{q_{t}-q_{md}}{q_{0}-q_{md}}\right]=-K_{d}\cdot t$$

where *K_d_* is the deswelling rate constant, *q_t_* is the weight of the hydrogel at predetermined time *t*, *q*_0_ is the weight of the hydrogel in the equilibrium swelling state (*t* = 0), *q_md_* is the weight at the end of the deswelling process, and *t* is time [1]. The values of *K_d_* obtained from the slope of the plot ln[*q_t_* − *q_md_*/*q*_0_ − *q_md_*] vs. *t* are given in Table 1. Based on the presented results, it is evident that AuNRs synthesized by Method II had the greatest influence on the deswelling process and that their incorporation led to an increase in the rate of medium release from the polymer matrix. The sample with the highest degree of swelling and VPTT (AuNRs-PNiPAAm hydrogel nanocomposites synthesized by Method II) also exhibited the fastest deswelling rate at temperatures above the phase transition.

### 3.4. XRD Analysis

**Figure 6:**
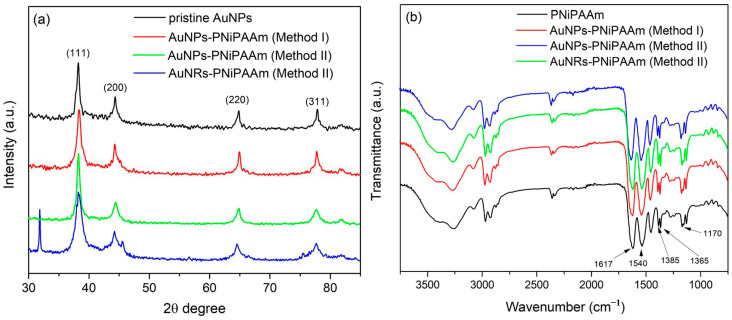
XRD patterns (**a**) and FTIR spectra (**b**) of nano Au-PNiPAAm hydrogel nanocomposites.

The crystalline nature of the AuNPs and AuNRs was confirmed by XRD analysis, and the obtained diffraction patterns are presented in Figure 6a. Since the chemically prepared nanoparticles synthesized by Method II were exposed to γ-radiation during formation of the polymer network, this analysis also could be used to evaluate how the absorbed doses affected the stability and structure of the nanoparticles. Four peaks obtained at 2θ around 38.3°, 44.6°, 64.6°, and 77.9° corresponded to the Bragg reflections from the (111), (200), (220), and (311) lattice planes, respectively, revealing that the gold nanoparticles possessed a well-defined atomic arrangement resembling the face-centered cubic (*fcc*) crystal structure occurring with bulk gold (JCPDS card no. 04-0784) [46].

The presence of clearly defined peaks of the crystal planes conclusively demonstrated that γ-radiation did not affect the nanocrystalline structure of the incorporated nanoparticles during the polymer matrix formation process (Method II). The average crystalline domain size was calculated according to Scherrer’s line-broadening equation, using the width of the (111) peak (JCPDS card no. 04-0784), as follows:

(14) $$D_{Sch}=\frac{k_{c}\lambda }{\beta cos\theta }$$

where *k_c_* is a numerical factor of the cubic structure (0.9), *λ* is the wavelength of the X-rays (0.1541 nm), *β* is the width (full-width at half-maximum) of the X-ray diffraction peak in radians (FWHM), and *θ* is the diffraction angle. According to these calculations, the average diameter for the colloidal pristine AuNPs was found to be 13.4 nm. Furthermore, values of 12.3 nm and 14.2 nm were obtained for the AuNPs (c = 2.5 × 10^−4^ mol/dm^3^) synthesized by Method I and Method II, respectively. Comparing the diameters of the AuNPs calculated from the UV–Vis spectra and XRD patterns, both calculations gave approximately the same values, with the slightly larger particles incorporated into the AuNPs-PNiPAAm hydrogel nanocomposites obtained by Method II.

### 3.5. FTIR Spectroscopy

FTIR spectroscopy was used to examine the molecular structure of the AuNPs-PNiPAAm and AuNRs-PNiPAAm hydrogel nanocomposites, and the resulting spectra are shown in Figure 6b. The used technique could reveal the nature of the electronic interactions between the incorporated nanoparticles and specific groups of polymer chains. The characteristic spectral peak of the NiPAAm monomer at 960 cm^−1^, which is associated with C=C bending and the existence of the vinyl group, disappeared in all presented spectra, indicating successful polymerization of the monomer [47]. The wide band in the range of 3700–3100 cm^−1^ corresponded to the secondary amide N–H stretching vibration of PNiPAAm. Two typical bands around 1385 cm^−1^ and 1365 cm^−1^ were related to the C-H stretching vibrations from the isopropyl group. The strong band in the range between 1617 and 1635 cm^−1^ was assigned to the characteristic C=O vibration in the amide I band of the NIPAAm molecule, while the amide carbonyl II band including N-H vibration was observed around 1540 cm^−1^. Asymmetrical C–H stretching was obtained in the range of 3000–2870 cm^−1^, whereas symmetric C–H stretching was obtained between 2400 and 2100 cm^−1^. The peak around 1170 cm^−1^ was derived from the amide III band of PNiPAAm. All of the above-observed peaks corresponded to the significant groups associated with the PNiPAAm chemical structure [2,47,48,49]. From the presented spectra, it could be noticed that the amide carbonyl I band shifted from 1617 cm^−1^ (PNiPAAm) to 1635 cm^−1^ (AuNRs-PNiPAAm), probably due to a donor–acceptor type of interaction, as was previously discussed [50]. In addition, the same spectral features of the PNiPAAm and AuNPs-PNiPAAm (Method I) nanocomposites confirmed that γ-irradiation during *in situ* AuNPs synthesis did not affect the macromolecular structure of the polymer matrix.

### 3.6. Thermo-Switchable Electrical Conductivity

Electrochemical reactions at the electrode–electrolyte interface involve multistep processes, such as mass transport, charge transfer, and adsorption, each occurring at distinct rates and timescales. To understand these time-dependent mechanisms, techniques like EIS are crucial. Because of its steady-state nature, small signal analysis, and the broad frequency range it covers (from less than 1 mHz to over 1 MHz), EIS stands out among electrochemical techniques for investigating the conductivity of complex systems like hydrogels. By exposing the hydrogel to electric stimuli that vary in frequency, EIS examines the charge transfer between electrodes and electrolyte. This technique employs a low-amplitude sinusoidal excitation signal, either in potential or current, and observes the resulting sinusoidal output signal. Commonly, the electrical current is measured when a potential difference is applied across two electrodes.

**Figure 7:**
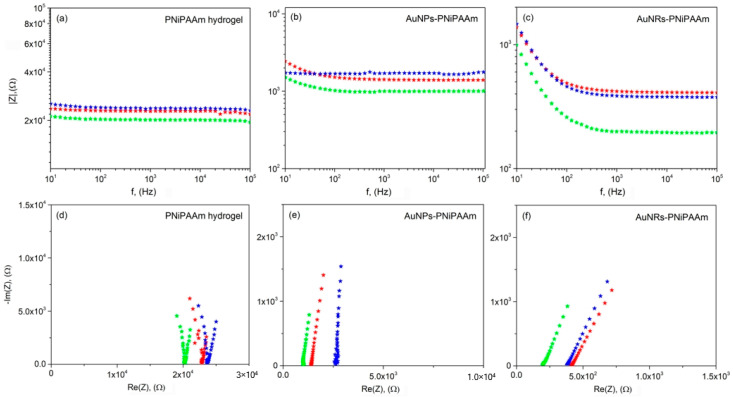
Bode plots of the impedance magnitude (**a**–**c**) and Nyquist plots for the real and the imaginary impedances (**d**–**f**) for PNiPAAm hydrogel and AuNPs-PNiPAAm and AuNRs-PNiPAAm hydrogel nanocomposites (c(NPs) = 2.5 × 10^−4^ mol/dm^3^) obtained by Method II (• Initial T = 25 °C, • T = 40 °C, • Final T = 25 °C).

In EIS, the sample’s response is expressed as impedance (*Z = Z*’ *+ Z*”), where *Z*’ represents resistance (real part) and *Z*” characterizes a reactive term showing ion movement delay in the system (imaginary part). However, this relationship is complex, influenced by various parameters that control the measured resistance. These aspects involve the bulk resistive-capacitive effects and electrode reactions, including adsorption or chemical modifications caused by reactive ion species and other variables. To minimize reactive effects, inert Pt electrodes are commonly employed, thus reducing chemical interactions between the sample and electrodes [51,52,53]. Figure 7 illustrates the Bode plots and Nyquist plots at various temperatures for the hydrogel nanocomposites containing gold nanoparticles of different shapes. Bode and Nyquist representations are widely employed for preliminary system analysis, assisting in identifying elementary processes within the mechanism.

**Figure 8:**
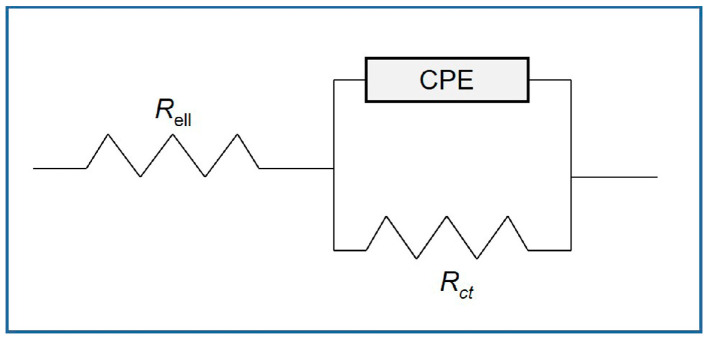
An equivalent electrical circuit used for the impedance plot fitting of the hydrogel nanocomposites.

A typical Bode plot provides an intuitive depiction of the impedance change with frequency. Figure 7a–c depicts the impedance of the hydrogels swelled in pure water in the frequency range of 10 Hz to 100 kHz. Within this frequency range, the nano Au-PNiPAAm hydrogel nanocomposites demonstrated a frequency-dependent response at low frequencies and frequency-independent behavior at high frequencies. The Nyquist diagram (Figure 7d–f), representing the imaginary (*Z*”) and real (*Z*’) components of impedance for each frequency, is another common representation that provides insight into the possible mechanism in an equivalent circuit model system [53,54,55]. By employing a fitting procedure on the acquired EIS spectra, the resistance values (*R*) of the hydrogel nanocomposites were determined. The collected EIS data were fitted using a Randles-type equivalent electrical circuit (Figure 8) with electrolyte resistance (*R_ell_*) coupled in series with a parallel combination of constant phase element (CPE, representing the double layer capacitance of the electrode) and faradaic/charge-transfer resistance (*R_ct_*).

**Figure 9:**
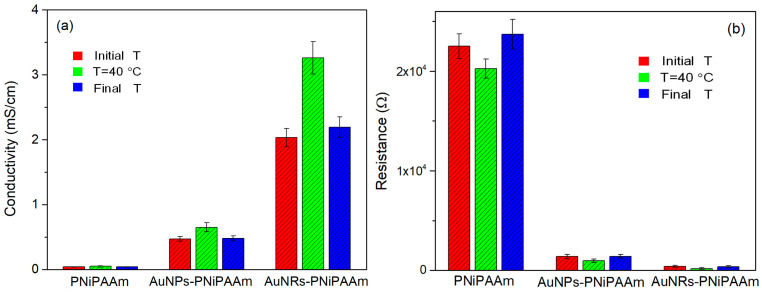
The electrical conductivity (**a**) and resistance (**b**) of PNiPAAm hydrogel and nano Au-PNiPAAm hydrogel nanocomposites.

The hydrogel in this setup served as a solid-state electrolyte, so the value of the electrolyte resistance, *R_ell_*, was taken as the resistance of the sample. The conductivity (*κ*) was determined by the following relationship:

(15) $$\kappa =\frac{R_{ell}\cdot l}{S}$$

where *l* is the thickness of the hydrogel sample, and *S* is the cross-sectional area of the hydrogel sample. The comparative analysis of all three samples showed that the pure PNiPAAm hydrogel (control sample) exhibited low conductivity and high resistance at the monitored temperatures (Figure 9). On the other hand, an increase in conductivity was observed for the nanocomposite samples, which pointed to the beneficial effect of the incorporation of gold nanoparticles into the polymer matrix to obtain conductive materials. Furthermore, an additional increase in conductivity of the nano Au-PNiPAAm hydrogel nanocomposites was noticed at the temperature above the phase transition temperature. Namely, in the transition from the fully swollen to collapsed hydrogel state, the electrical conductivity of the sample with nanospheres slightly increased from 0.47 to 0.65 mS/cm, while in the case of the sample with nanorods, the conductivity was significantly boosted from 2.03 to 3.26 mS/cm (Figure 9a). This increase in electrical conductivity was caused by temperature-dependent changes in the polymer matrix. Above the VPTT (at 40 °C), the polymer chains of PNiPAAm collapsed, changed conformation, and thus reduced the swelling capacity. Therefore, the incorporated nanoparticles were at a shorter distance, causing facilitated charge transfer and an increase in electrical conductivity. At the same time, the impedance and resistance of the samples reach their lowest values (Figure 9b). The described trend was fully reversible, meaning that the return to room temperature led to an increase in resistence and a decrease in conductivity. Regardless of nanoparticle shape, this change was observed in both samples, with nanospheres and nanorods. This remarkable “on–off” thermo-switchable mechanism was attributed to the sensitive nature of the thermo-responsive PNiPAAm chains [11]. By comparing the samples containing the same concentration of nanospheres or nanorods, it was apparent that the hydrogel nanocomposite with rod-shaped gold nanoparticles had much greater electrical conductivity.

According to the literature, the conductivity of nanocomposites can be influenced by the nanoparticle concentration, size, shape, dielectric properties, and local environment [56]. The variation in conductivity of the investigated hydrogel nanocomposites could be attributed to the varied geometry of the nanoparticles as well as the local environment. Namely, at lower temperatures, the highly swollen hydrogels hindered connectivity amongst the incorporated nanoparticles, disabling charge transfer through the hydrogels, thus resulting in low conductivity. At temperatures above the VPTT, the shrinking of the hydrogels facilitated connectivity between nanoparticles, leading to increased electrical conductivity. This effect was more pronounced for nanorods due to their length (aspect ratio) and the formation of a percolated network of electrical contacts amongst the incorporated nanoparticles, providing pathways for electrons to travel [57].

## 4. Conclusions

In this study, thermo-responsive and conductive hydrogel nanocomposites based on poly(*N*-isopropylacrylamide) and gold nanoparticles (nanospheres and nanorods) were produced using different synthesis methods. Comparing the two applied synthesis procedures, it was found that Method II (combination of chemical and radiolytic processes) was more suitable for obtaining hydrogel nanocomposites that were stable for a long period of time. The TEM analysis revealed the formation of nanospheres with a mean diameter of around 17 nm and nanorods with an aspect ratio of around 4.7 (≈8 nm in diameter, and ≈37.5 nm in length). The morphology and face-centered cubic crystal structure of the nanoparticles were confirmed by the UV–Vis absorption spectra and XRD analysis, respectively. The incorporation of AuNRs had a greater influence on the physicochemical parameters of the hydrogel nanocomposites, causing a higher swelling capacity and diffusion coefficient as well as increasing the VPTT to slightly higher values. The results of the EIS measurements indicated that the incorporation of both AuNPs and AuNRs increased the electrical conductivity, with a much more pronounced effect in the case of AuNRs. Moreover, the investigated nano Au-PNiPAAm hydrogel nanocomposites showed temperature-dependent electrical conductivity below and above the VPTT. The existence of a fully reversible “on–off” thermo-switchable mechanism, regardless of nanoparticle shape, made these systems especially suitable for potential use as sensors. Thus, the obtained nano Au-PNiPAAm hydrogel nanocomposites belonged to electrically conductive polymeric materials that possess flexibility (originating from hydrogel matrix) and electrical conductivity (originating from gold nanoparticles as conductive component). Moreover, the use of γ-radiation enabled synthesis and sterilization in one technological step, which is crucial for potential biomedical applications of these systems. By summarizing these findings, it is possible to propose a future research direction for the fabrication of nanocomposite materials with integrated multifunctionality.

## Figures and Tables

**Table 1:** Physicochemical parameters of investigated samples.

	c(NPs) × 10^4^ (mol/dm^3^)	SD_eq_	n	D × 10^7^ (cm^2^/s)	K_d_ × 10^3^ (1/min)	VPTT (°C)
PNiPAAm hydrogel	0	12.6 ± 0.31	0.51 ± 0.01	1.5 ± 0.030	5.1 ± 0.15	30.1 ± 0.5
*Method I*						
AuNPs-PNiPAAm	1.0	15.2 ± 0.38	0.59 ± 0.02	2.9 ± 0.033	11.8 ± 0.26	30.7 ± 0.7
	2.5	15.1 ± 0.37	0.57 ± 0.01	3.1 ± 0.034	11.2 ± 0.25	30.9 ± 0.6
AuNRs-PNiPAAm	1.0	12.9 ± 0.33	0.56 ± 0.01	2.2 ± 0.027	5.0 ± 0.17	30.1 ± 0.5
	2.5	13.1 ± 0.32	0.54 ± 0.01	1.8 ± 0.022	5.9 ± 0.19	30.2 ± 0.5
*Method II*						
AuNP-PNiPAAm	1.0	19.0 ± 0.48	0.58 ± 0.01	3.14 ± 0.028	12.1 ± 0.25	31.0 ± 0.7
	2.5	18.5 ± 0.49	0.61 ± 0.02	5.81 ± 0.039	10.7 ± 0.27	31.1 ± 0.6
AuNRs-PNiPAAm	1.0	20.1 ± 0.57	0.64 ± 0.02	7.73 ± 0.049	11.9 ± 0.25	32.5 ± 0.8
	2.5	25.8 ± 0.65	0.69 ± 0.02	8.44 ± 0.053	12.3 ± 0.28	32.6 ± 0.7

## Data Availability

The original contributions presented in the study are included in the article and further inquiries can be directed to the corresponding author.
